# Fathering style influences health outcome in common marmoset (*Callithrix jacchus*) offspring

**DOI:** 10.1371/journal.pone.0185695

**Published:** 2017-09-28

**Authors:** Toni E. Ziegler, Megan E. Sosa, Ricki J. Colman

**Affiliations:** 1 Wisconsin National Primate Research Center, University of Wisconsin, Madison, Wisconsin, United States of America; 2 Department of Cell and Regenerative Medicine, School of Medicine and Public Health, University of Wisconsin, Madison, Wisconsin, United States of America; University of Tasmania, AUSTRALIA

## Abstract

In the cooperative breeding common marmoset monkey, *Callithrix jacchus*, fathers share the care responsibility and energetic load with their mate from the time their infants are born. However, not all fathers show the same level of participation in direct infant care. Here we present the first results demonstrating that fathering style can improve both survival and growth trajectory of a male’s offspring during the first 30 weeks of life and that these infant outcomes are consistent within a father throughout successive births. Twenty-four marmoset fathers were tested for their responsiveness to an infant distress call when their infants were approximately two weeks old. These fathers were categorized as either responsive (RS) or nonresponsive (NRS) based on their response to the calls. Survival past 1 month was then determined and bi-monthly weights of current infants through 30 weeks of age were taken. Infant survival to the first month was significantly higher with RS fathers than with NRS fathers during this critical time period. Infants from RS fathers also had a higher growth trajectory with significant differences in body weight in the 28^th^ and 30^th^ week after birth. Only the RS fathers showed a significant increase in serum testosterone in response to infant cries suggesting a physiological role of testosterone in the motivation to search for the infant. Furthermore, all offspring born to RS fathers from subsequent births also showed a significantly higher survival rate and higher growth trajectory than for offspring of NRS fathers. These results suggest that fathering style is a consistent trait and responsive fathers improve infant survival rate and growth during the first 30 weeks. Such fathering style traits may be passed on to the male offspring due to environmental or genetic factors.

## Introduction

Mammals are born vulnerable to their environment, needing protection and direct care to survive. At birth, maternal care is stimulated through sensory stimuli and endogenous endocrine changes, and in most species, it is the sole responsibility of the mother to ensure offspring survival. However, in some mammalian, avian and invertebrate species males or other group members remain to assist the mother in infant care and take some of the responsibility for offspring survival. In these cooperative breeding systems the costly energetics of care can be shared, benefiting the mother with higher reproductive activity and increasing production of energetically costly offspring [1, 2]. Cooperative breeding structures may take many forms including the presence of older offspring of the breeding pair or groups with multiple breeding males and females available to provide supportive care for the infant(s) [3].

Due to the extended time to adulthood in primates, long-term parental investment is required, and therefore, alloparenting is common, especially in humans [4]. The primate family Callitrichidae demonstrate a highly specialized cooperative breeding system in which the breeding male provides the majority of the infant care and older offspring remain in the social group past puberty to learn and assist with infant care. Although these cooperative breeding strategies evolved independently from human strategies, Callitrichid monkeys are an excellent model for understanding derived life history and demographic traits involved in cooperative care [5 6,7,8].

The common marmoset, *Callithrix jacchus*, provides allocare for the newborn(s) immediately after birth [9]. Older siblings learn parenting skills by caring for their younger siblings [10]. However, during the first critical weeks it is mainly the father who shares the burden of carrying infants with the mother. Common marmoset fathers begin to carry infants on the day of their birth [11,9] and will carry their infants an average of 50% of the time during the first week [9]. Fathers show the highest levels of grooming and licking the newborn in the first weeks after birth even though others are available for allocare [12]. Due to the mother’s rapid postpartum ovulation (within 10 to 20 days) with resultant pregnancy, as well as having multiple infants at birth, fathers are needed to share in the energetic burden in both wild and captive marmosets [13,14]. Benefits for biparental fathers include increased offspring survival, female fecundity, and in turn, increasing inclusive fitness to the male leading to an evolutionary feedback between male care and life history traits [15]. Additionally, marmoset fathers benefit by having an active hormonal response to the female’s pregnancy and to his offspring in the early postpartum period where males show increased levels of the metabolic hormone prolactin in response to infant stimuli, that ultimately maintains a father’s weight while actively carrying infants [14].

While common marmoset fathers often participate extensively in infant care, individual participation between males varies greatly [11,16]. Using a motivation test specifically designed to determine the fathers’ motivation to respond to an infant, common marmoset fathers varied in their motivation to respond to recorded distressed infant cries or live infants [9,14]. Experienced fathers were significantly more responsive to infant cries than paired males who had not been fathers, but only ~60% of the experienced fathers were responsive in the motivation test [16]. When treated with estradiol, responsive fathers increased their responsiveness to the infant distress calls but non-fathers did not increase responsiveness indicating estrogens can only enhance parental motivation in an experienced father but not initiate the onset of parenting in male marmosets [17]. This test correlates with the father’s carrying behavior and time spent with offspring in the first few weeks after birth only when interference by other allocare family members does not occur [9].

Marmosets wean infants by ages 5–10 weeks. This is a critical time when mothers are rejecting their offspring more often and fathers are encouraging the introduction of food. While both parents transfer food to infants during weaning, fathers are the first and most responsible for introducing solid food during weaning [18, 12]. Thus, marmoset fathers appear to have an important role in ensuring the survival and health of their offspring. Male care in biparental mammals may therefore benefit the offspring by increasing their survival to independence including increased growth rates or size [17]. However, such definitive data on infant outcomes for biparental primates does not exist.

Human societies have cultural influences and changing economic conditions that have led to increased paternal involvement in the early care of offspring yet little is known about the impact of the father’s parenting style on child development. Direct involvement of human fathers has been associated with positive infant physical, cognitive and social development including improved weight gain, higher breastfeeding rates, higher receptive language skills and higher academic achievement. However, the data is qualitative, using self-reports of the father-infant interactions [19]. Similarly, preterm infants show enhanced cognitive skills with high-involvement of fathers, but again the data are based on maternal recording of the father’s involvement [20]. One previous study in common marmoset monkeys examined early rearing effects on physical growth and found that marmoset infants who were abused (parents pushing the infants off, biting them and ignoring their cries) had a significantly lower body weight after 10 weeks of age than infants that were not abused [21]. However, few other studies have focused on the social, physical or neurobiological development of offspring related to a father’s motivation to provide parental care.

Since marmoset fathers vary in responsiveness to their offspring, we can test if rearing by responsive fathers increases survival and health benefits to offspring. If fathering style is important, then we expect to see that fathers who are responsive to infant distress cries will be more responsive to their own offspring in the early weeks of care through to weaning thereby providing an increased chance of survival and increased growth trajectory (Experiment 1). We will also test to see if there is a physiological response to the infant cries. If responsiveness to offspring cries is a consistent trait, then we expect offspring of responsive males to have better health outcomes than offspring of nonresponsive fathers. Therefore, we predict that males who are responsive to infant cries will have a higher percent survival rate and increased growth rates for all their offspring when compared to males who do not respond to infant distress calls (Experiment 2). Additionally, we compared the amount of older offspring available to help with the infants to determine if the effect of having older siblings in the group could explain the higher survivability.

## Materials and methods

### Ethics statement and animal care

All marmosets assigned to this study were housed at the Wisconsin National Primate Research Center at the University of Wisconsin-Madison, an AAALAC accredited facility that meet USDA standards. The Graduate School Animal Care and Use Committee (ACUC) monitors the housing and animal condition regularly to ensure all guidelines are met for the safety and health of the marmosets. This NIH funded study (HD057684) was reviewed and approved by the University of Wisconsin Graduate School ACUC, G00526. Experiments were conducted in accordance with international standards on animal welfare and were compliant with national regulations. As per the ACUC approved protocol, all marmosets were observed at least twice per day to monitor health. Any signs of pain were treated with an analgesia from the following options: ketprofen, meloxicam, or an alternative, if recommended by a veterinarian. None of the studies required endpoints of euthanasia. If an animal was sick and required euthanasia a veterinary approved general anesthesia followed by an IV overdose of sodium pentobarbital or equivalent was approved in the ACUC protocol.

Marmosets used for this study were housed in family cages varying in size between 0.61 x 1.22 x 1.83 m to 0.6 x 0.91 x 1.83 m and including branches and appropriate vertical space for movement [16]. The colony is maintained on a 12-h light:dark cycle (6:30–18:30 light), a steady temperature of 27°C, and humidity approximately 49%. Marmosets are fed twice daily at approximately 0800 h and 1300 h in standardized meals consisting of marmoset chow (Mazuri 5M16, LandO’Lakes) and supplemental fruit, mealworms, and vegetables. Water is provided *ad libitum*.

### Subjects

Infant survival and weight across the early development period were determined for offspring from 24 common marmosets that had been previously identified as either responsive (RS) or nonresponsive (NRS) fathers to infant distress cries in a motivation test [14,16]. Survival and weight data were obtained from the Wisconsin National Primate Research Center’s Electronic Health Record database. The infant mortality rate for the colony is near 50% including stillbirths but near 35% without including stillbirths. This is similar to what other marmoset colonies report as the increased number of triplet and quadruple births also increases the percentage of mortality.

To test father’s responsiveness to the infant distress cry, fathers were placed in a separate room in a cage similar to their home cage, while a 10-minute recording of a novel marmoset infant distress call (1–2 week-old infant) or a vocal control was played in an adjoining cage. In order to respond to the distressed calls, the father had to cross a mesh bridge and enter the cage where the infant sounds were located [9]. Based on whether they crossed the bridge and investigated the source of the infant cries, fathers were divided into either RS (fathers who responded to the stimulus within 10 minutes, n = 15) or NRS (fathers who never investigated the stimulus source, n = 11). The infant distress calls were pre-recorded and included an average of 69 vocalizations/minute as reported previously [9,16]. There was no increased mortality of infants used in these studies as any infant death occurred prior to father testing and was incidental. Most of the infants died within the first week after birth.

### Experiment 1

In experiment 1, infant survival and growth trajectories were determined for the offspring of 24 males who were previously determined to be either RS or NRS to infant distress cries. All males were experienced fathers and had at least one previous birth with his mate. These 24 males experienced births of single, twin and triplet births resulting in 62 offspring, 39 of whom survived (Table 1). The survival of offspring was determined by who survived past the first month. All infants were weighed regularly from 2 weeks of age to 30 weeks of age. The number of older offspring present at birth was also recorded for each group to determine if this explained some of the difference between the two groups.

**Table 1:** Number of births resulting in singleton, twins and triplets and percent survived by birth type for RS and NRS fathers.

Birth type	RS Fathers (n = 15)	% Survived	NRS Father (n = 9)	% Survived
Singleton	2	100	0	N/A
Twins	8	95.5	4	80
Triplets	5	80	7	38
Total	15		11	

Within 5 minutes after completion of behavioral testing (as described under subjects above), 0.5 – 1ml of blood was collected from the femoral vein while animals were awake and manually restrained. All animals had been previously habituated to the restraint system. Males were returned to their family directly after blood sampling. Father responsiveness to infant distress cries and blood sampling were performed as part of a previous study. We opportunistically measured serum testosterone in all samples available to us (4 RS and 4NRS fathers) in response to a control vocalization and at another time to an infant vocalization. For the testosterone assay, 15 μl were taken, diluted to 500 μl and extracted with 5 mls of diethyl ether. Samples were dried and re-suspended in assay buffer and analyzed by an in-house enzyme-immunoassay as described in Ziegler et al. [22].

### Experiment 2

To determine if parenting style is a consistent trait, each infant born to the fathers from experiment 1 was assessed for their survival and weight gain as described above. Infants from the first birth for each male were excluded from this experiment as it is well known that infant survival rate is poor for first-time common marmoset parents [10]. The entire number of infants per birth born during all recorded births for fathers who have been categorized as RS or NRS fathers is listed in Table 2. The number of offspring used for weight data per father ranged from 3 to 12 (mean = 7; median = 8).

**Table 2:** The entire number of births occurring as single, twin, triplet and quadruplet during all recorded births for fathers who have been categorized as RS or NRS fathers.

Number of infants	Responsive fathers	Nonresponsive fathers	Total
Single	18	14	**32**
Twin	30	13	**43**
Triplet	45	28	**73**
Quadruplet	2	7	**9**
**Total**	**95**	**62**	**157**

### Data analyses

#### Experiment 1

We determined the relationship between the number of previous births to each father and the fathers’ classification as RS or NRS by unpaired t-test. We similarly used an unpaired t-test to compare body weights of RS and NRS fathers at the time of their infants’ birth. Difference in infant survival for RS versus NRS males was evaluated by Fisher’s exact test. For purposes of this study, infants who survived the first month were determined to have “survived”; those who died within the first month following birth were determined to have “died”. Difference in infant weight at two weeks of age between RS and NRS fathers was determined by unpaired t-test. For each infant, all available weights between 2 and 30 weeks of age were used to generate a linear regression line specific to their weight change over time. This resulting regression equation was then used to generate weights at 2-week intervals. To determine if the offspring growth trajectories were different by condition, we performed a 2-way ANOVA for time and condition with Bonferroni post-hoc correction. We evaluated weight from birth up to 30 postnatal weeks for two reasons. First, the father’s involvement in parenting peaks during this period, and second, new infants are likely born into the family group around this time leading to a change in parenting focus. The number of older siblings present at the time of birth per group was compared by Mann-Whitney U test between the RS and NRS fathers. Percent change in testosterone concentrations from control to infant distress vocalizations were compared by two-tailed Mann-Whitney U test between RS and NRS fathers.

The testosterone data for the infant distress cries condition was converted to percent change from the testosterone values for the vocal control condition. A Mann Whitney test was performed to determine significant differences between the RS and the NRS condition. Results were considered significant at p<0.05.

#### Experiment 2

Survivorship, infant weight at two weeks of age, and weight trajectory were determined as in Experiment 1. The RS fathers had an average and range of 6.54 (2–13) offspring per father used for this assessment while the NRS fathers had an average and range of 13.89 (9–34) offspring per father. For each father, we averaged the weights of their offspring at each 2 week period to provide the average offspring weight per father at each time point in order to perform the Repeated Measures Mixed Model ANOVA for time and condition with Bonferroni post-hoc correction.

## Results

### Experiment 1

**Fig 1:**
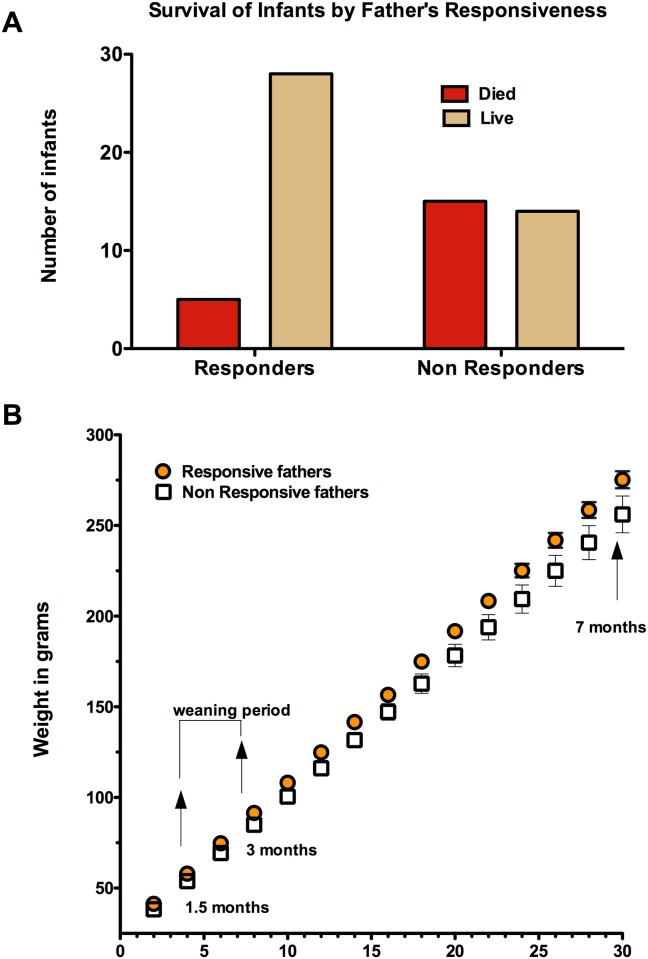
A. Experiment 1. Infant survival is significantly higher for common marmoset fathers who are responsive to infant distress cries (RS) compared to fathers who show no response to infant distress cries (NRS), p = 0.002. Red bars indicate the number of infants that died while the brown bars indicate the number of infants that lived. B. Experiment 1. Mean linear growth curves for infant marmosets under two conditions: RS and NRS fathers. Weights between the two conditions began to separate during the weaning period and are significantly (*p < 0.05) different by weeks 28 and 30.

Classification as RS or NRS was not significantly related to how many previous births the fathers had experienced (t = 0.78, p = 0.44, two-tailed) indicating that this was not an experiential effect. No differences were found between RS and NRS father’s weights at the time of their infant’s births (t = 0.68, p = 0.50, two-tailed). Infants born to RS fathers had a significantly improved survival rate past 1 month of age compared to infants of NRS fathers (p = 0.002, n = 62 infants; Fig 1A).

Infant weight at two weeks of age did not differ by condition (offspring of RS fathers = 41 ± 1.5 grams, offspring of NRS fathers = 38.0 ± 1.4 grams; t = 1.3, p = 0.19). As expected, infant weights in both conditions increased with time (F = 1869.57, df = 14, 546, p < 0.001). Over the first 30 weeks, there was a significant interaction between time and condition for infant body weight (F = 2.40, df = 14, 546, p = 0.003) and differences between the RS and NRS condition (F = 5.21, df = 1, 39, p = 0.03). Mean body weights of the groups began to separate during the 6–12 week weaning period (Fig 1B) and became significant at weeks 28 and 30.

No difference was found between the RS and the NRS conditions by number of older siblings present per birth (U = 64, p = 0.74). The number of older siblings per birth ranged from 0 to 4 for the RS condition and 0 to 6 for the NRS condition.

**Fig 2:**
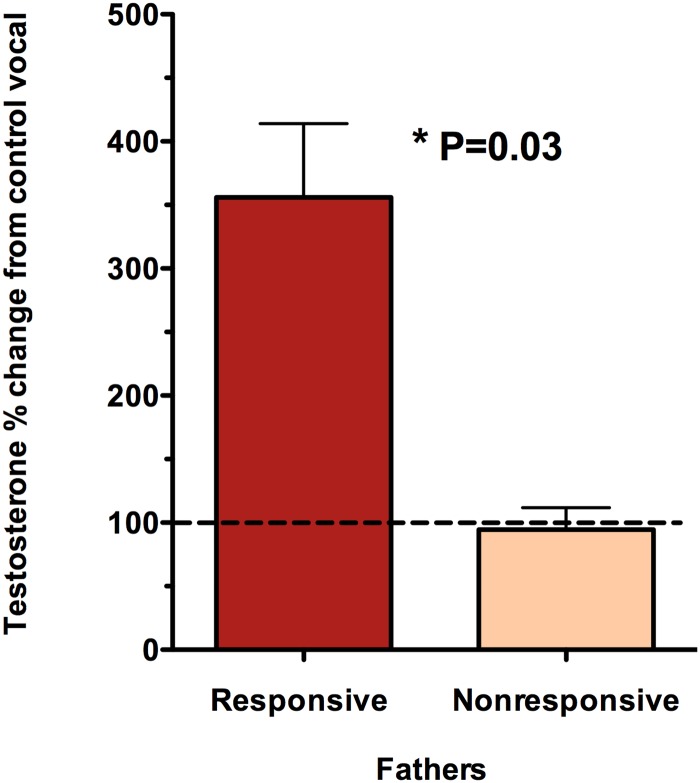
Experiment 1. Testosterone percent change from control vocal to infant distress cries. RS fathers showed a significant change (p = 0.03) in their testosterone levels after searching for the source of the infant cries while NRS fathers did not show any testosterone change.

Fig 2 shows the percent change of testosterone levels from the control vocalization to the infant distress cries condition. No change was observed for the NRS males but the RS males showed a significant increase in testosterone after searching for the source of the infant cries (sum of ranks RS = 26, NRS = 10, U = 0.0, p = 0.03). RS males had low testosterone in the control vocal test that increased significantly after the infant vocal test indicating that the RS fathers were reacting physiologically to the distress cries. This response was independent of the age of the father’s own offspring (2 weeks to 5 months of age).

### Experiment 2

**Fig 3:**
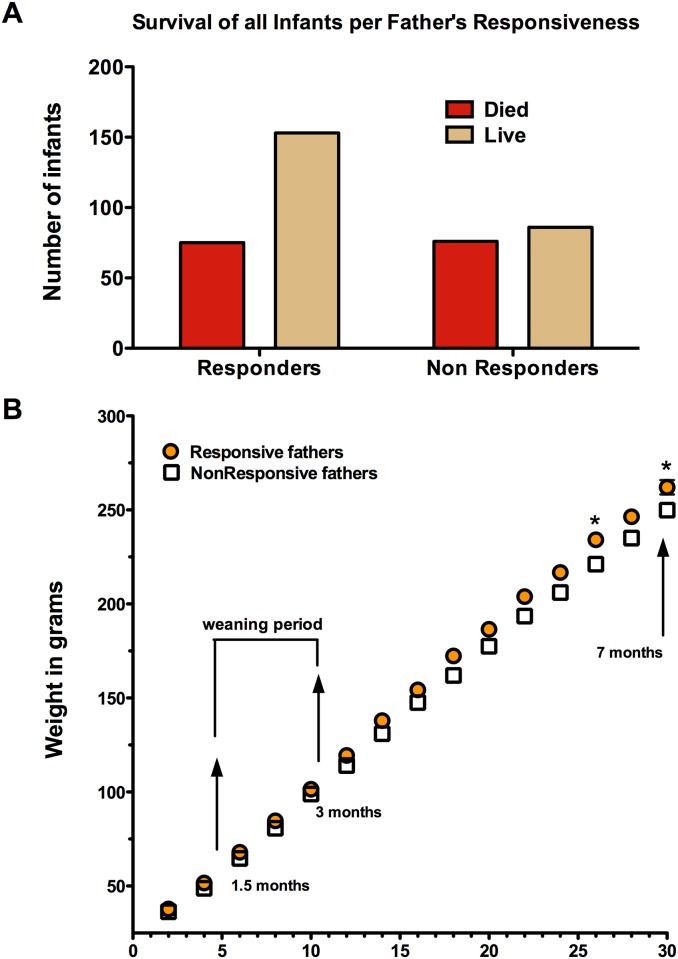
A. Experiment 2. All offspring of RS fathers have a higher survival rate than offspring of the NRS fathers, P = 0.006. B. Experiment 2. The mean weights for each father’s offspring over collective births for RS or NRS fathers. The two conditions were significantly different at 26 and 30 weeks (p < 0.05).

To determine if fathering style (RS or NRS) was a consistent trait within each male, the survivorship and weight trajectory for all offspring from each father evaluated in Experiment 1 were determined. As in Experiment 1, infants born to RS fathers had a significantly improved survival rate past 1 month of age compared to those born to NRS fathers (p = 0.006, n = 390 infants; Fig 3A). Infants from RS fathers had a 67% survival rate while infants from NRS had a 53% survival rate.

Infant weight at two weeks of age did not differ by condition (RS offspring = 37 ± 0.72 grams, NRS offspring = 37.0 ± 2.4 grams; t = 0.43, p = 0.67). As expected, infant weights in both conditions increased with time (F = 1019.61, df = 14, 330, p < 0.0001). An interaction was found between time and condition (F = 2.59, df = 14, 280, P = 0.002). As expected, time accounted for 89.65% of the total variance (F = 3458.87, df = 14, 280, p < 0. 001) and condition was significant (F = 5.64, df = 1, 20, p = 0.03). Following post hoc Bonferroni correction infant weights at weeks 26 and 30 were significantly higher for the RS compared to the NRS condition (p < 0.05), Fig 3B.

## Discussion

We found significant improvement in infant survival in the critical first month of life when fathers were responsive to infant needs. Offspring of RS fathers also had a significantly higher weight trajectory starting after weaning when compared to offspring of NRS fathers. Without direct care in the first few weeks of life marmoset infants are vulnerable due to lack of thermoregulation, predators in the wild, physical injuries, and psychosocial effects on their growth and development. The quality of parental care in the common marmoset therefore can influence later growth and behavior [21], and so fathering style, as has been observed with mothering style, may have profound effects on health outcomes of offspring. Furthermore, we provide evidence that all offspring from responsive fathers have increased survival and weight gain suggesting that paternal style is a consistent trait in marmosets.

The family Callitrichidae encompasses mainly species that produce multiple offspring at birth. Births occur as singleton, twins, triplets and even quadruplets as females produce multiple ovulations within a single ovulatory cycle [23,24]. While marmosets are highly fecund, the percentage of infant survival is extremely variable [25]. It has been suggested that infant survival is related to population density, availability of food, birth season, infant birth weight, litter size and the social environment [26]. Birth weight is inversely influenced by litter size when the number of infants at birth is more than two. In Experiment 2, we found that RS fathers had more offspring survive in triplet births (83%) than did NRS fathers (27%). This provides evidence that social environmental factors may have a big impact on the number of surviving infants. Not only do the RS fathers provide benefits to their offspring but to their mate as well; the mother could not ensure survival of triplets and conceive again during the postpartum ovulation without a responsive mate. We did not find any evidence that the number of older siblings influenced the survival outcome of the infant. While allocare is important in the marmoset cooperative breeding social organization, the father is the primary caretaker of infants during the critical first week. The older siblings increase their interactions with the newborn after this week and it is the male siblings that are the most interactive with the infants [12].

While we found no differences in marmoset infant body weights by condition at two weeks of age, infants of RS fathers began to show higher mean weights during weaning. By the end of the weaning period the mean weight was clearly higher for infants in the RS condition than for those in the NRS condition. This trajectory continued through the thirty weeks of study becoming significant at the last two time points (weeks 28 and 30). Other studies at different marmoset colonies have noted differences in offspring weight gain but have looked at different factors. Johnson [21] suggested that the frequency of positive parental behaviors during infancy is correlated with stature when the monkeys reach 10 and 20 weeks of age. Infants that were mistreated by their parents during infancy were smaller in body weight [21]. Following weaning at around 10 to 12 weeks, their profiles showed a divergence in the weights with normal infants weighing more and abused infants having a slower growth trajectory. This was consistent with the profiles seen in our study although our NRS condition was more similar to neglect than abuse. Another study indicated a correlation between higher suckling frequencies and increased growth rate but early consumption of solid food was negatively associated with growth and weight during the following month [25]. Tardif [25] suggests that earlier, more frequent, independent locomotion in the common marmoset may be a stressor affecting subsequent growth via either psychosocial or nutritional stress. Based upon these studies and our data, we suggest that fathers who are responsive to infant distress calls are more responsive to infant’s needs, enabling infants to access their mothers for nursing more readily and transitioning their offspring to solid food at the appropriate time.

Common marmoset fathers are highly involved in the weaning process by transferring food to their offspring or responding to infant begging calls for food during the weaning transition [18]. While mothers and fathers are equally involved in the weaning process, at the start of weaning, mothers have usually conceived again requiring fathers to share the energetic demands of weaning infants during this high growth time. In fact, data on families’ food sharing at weaning indicates that fathers are the first to initiate this important milestone in a marmoset’s development [12]. While our study did not collect data at weaning time for the offspring, it is likely that RS fathers were more highly involved in the initiation of weaning than NRS fathers.

The use of infant distress calls as an indicator of father’s motivation to parent has been used not only for marmoset fathers [9, 14] but also for humans. Distress cries are the main communication newborns have and are essential for initiating a parental response [27]. Human fathers are just as good as mothers at recognizing the cries of their babies as long as they have time to spend with their baby [28]. Fathers are more responsive to infant cries than non-fathers, and experienced fathers show the greatest increase in testosterone from baseline compared to nonfathers; i.e., they are more reactive [9,29]. In another human study fathers also showed increased testosterone levels with infant cries but their testosterone levels were lower when the baby cries were associated with the chance to show nurturing responses to the baby [30]. Marmoset fathers are also significantly more responsive to infant cries than non-fathers [9]. Marmoset fathers have low testosterone while they are caring for infants [14] and their infant’s scent alone can cause an acute drop in testosterone associated with increased estrogen production [31]. Elevated testosterone following a search of the source of infant cries, or the sound of cries alone may, as found in the current study, promote a protective father who is preparing to defend or protect their offspring who may be in danger [30]. While our sample size was small for the testosterone data, marmoset fathers varied in their behavioral and physiological responses to their infants potentially influencing their offspring’s overall health outcome. Variations in fathers’ attentiveness to their offspring is high both in marmoset and human fathers, highlighting the importance of understanding the neurobiology of optimal paternal responses to cries in cooperative breeding, biparental care species [16,32].

What is the mechanism that motivates fathers to be responsive to their infants needs and therefore provide the offspring with an advantage in life? It could be that those fathers who are in close contact with their mate and have a strong pair-bond prior to the birth will be in close contact to the offspring at birth and thereafter. In the common marmoset, Finkenwirth et al. [33] have shown that high pre-birth affiliation between the pair-mates leads to more caretaking when they are parents. The sensory contact with the infant then influences the father’s hormonal system thereby promoting father-infant bonding. Alternatively, the differences in fathers’ responsiveness to their infants could be due to genetic traits or environmental conditions that promote epigenetic changes in father motivation. Clearly in this study, fathers who were responsive to the infant cries at one birth were responsive for all of their infant’s births. Further research will be required to determine if these traits are generational.

The concept that fathers are important in the survival and wellbeing of their offspring is not novel. In the mammalian orders where cooperative care is common, fathers provide essential needs to the offspring. In humans, cross-cultural studies show that paternal care is widespread, and therefore, show the characteristics of the other cooperative breeding mammalian species [34]. A number of human studies have found that paternal care has effects on wellbeing and health [20,33,35]. Associations between quality paternal care and wellbeing are as important as maternal care at some ages in development [36]. Further, low father involvement in infant care has been linked with risk factors for later mental health symptoms [36,37]. The developmental-experiential process begins in infancy where children exposed to early adverse experiences, such as neglect are at increased risk for the development of depression, anxiety disorders, or both [38]. Therefore, quality involvement from both parents is essential for positive child affective development.

## Conclusions

Infants of marmoset fathers who actively search for the source of an infant distress call have significantly improved survival during the first month of life and a higher weight trajectory following weaning up to 30 weeks of age. Additionally, parenting style and associated infant outcome are consistent within individual fathers over multiple reproductive events. Our findings have broad implications for a fathering style that may be passed on to male offspring due to environmental or genetic factors.

## Supporting information

S1 Dataset Datasets for the statistical tests for the survival and weights for Experiment 1 and 2. (XLSX)
